# Clinical effectiveness of a sedation protocol minimizing benzodiazepine infusions and favoring early dexmedetomidine: a before-after study

**DOI:** 10.1186/s13054-015-0874-0

**Published:** 2015-04-02

**Authors:** Lee P Skrupky, Anne M Drewry, Brian Wessman, R Ryan Field, Richard E Fagley, Linda Varghese, Angela Lieu, Joshua Olatunde, Scott T Micek, Marin H Kollef, Walter A Boyle

**Affiliations:** Department of Pharmacy, Aurora Baycare Medical Center, 2845 Greenbrier Road, PO Box 8900, Green Bay, WI 54311 USA; Department of Anesthesiology, Washington University School of Medicine, 660 South Euclid Avenue, Campus Box 8054, St Louis, MO 63110 USA; Department of Emergency Medicine, Washington University School of Medicine, 660 South Euclid Avenue, Campus Box 8072, St Louis, MO 63110 USA; Department of Anesthesiology & Perioperative Care, UC Irvine Medical Center, 333 City Boulevard West, Suite 2050, Orange, CA 92868 USA; Department of Anesthesiology, Virginia Mason Medical Center, 1100 Ninth Avenue, Mailstop B2-AN, PO Box 900, Seattle, WA 98101 USA; Department of Anesthesiology, University of Wisconsin School of Medicine and Public Health, 600 Highland Avenue, B6/319 CSC, Madison, WI 53792 USA; Ernest Mario School of Pharmacy, Rutgers, The State University of New Jersey, 11 Country Squire Lane, Holmdel, NJ 07733 USA; 3540 Birchbark Drive, Florissant, MO 63033 USA; St Louis College of Pharmacy, 4588 Parkview Place, St. Louis, MO 63110 USA; Department of Pulmonary and Critical Care, Washington University School of Medicine, 660 South Euclid Avenue, Campus Box 8052, St. Louis, MO 63110 USA

## Abstract

**Introduction:**

Randomized controlled trials suggest clinical outcomes may be improved with dexmedetomidine as compared with benzodiazepines; however, further study and validation are needed. The objective of this study was to determine the clinical effectiveness of a sedation protocol minimizing benzodiazepine use in favor of early dexmedetomidine.

**Methods:**

We conducted a before-after study including adult surgical and medical intensive care unit (ICU) patients requiring mechanical ventilation and continuous sedation for at least 24 hours. The before phase included consecutive patients admitted between 1 April 2011 and 31 August 31 2011. Subsequently, the protocol was modified to minimize use of benzodiazepines in favor of early dexmedetomidine through a multidisciplinary approach, and staff education was provided. The after phase included consecutive eligible patients between 1 May 2012 and 31 October 2012.

**Results:**

A total of 199 patients were included, with 97 patients in the before phase and 102 in the after phase. Baseline characteristics were well balanced between groups. Use of midazolam as initial sedation (58% versus 27%, *P* <0.0001) or at any point during the ICU stay (76% versus 48%, *P* <0.0001) was significantly reduced in the after phase. Dexmedetomidine use as initial sedation (2% versus 39%, *P* <0.0001) or at any point during the ICU stay (39% versus 82%, *P* <0.0001) significantly increased. Both the prevalence (81% versus 93%, *P* =0.013) and median percentage of days with delirium (55% (interquartile range (IQR), 18 to 83) versus 71% (IQR, 45 to 100), *P* =0.001) were increased in the after phase. The median duration of mechanical ventilation was significantly reduced in the after phase (110 (IQR, 59 to 192) hours versus 74.5 (IQR, 42 to 148) hours, *P* =0.029), and significantly fewer patients required tracheostomy (20% versus 9%, *P* =0.040). The median ICU length of stay was 8 (IQR, 4 to 12) days in the before phase and 6 (IQR, 3 to 11) days in the after phase (*P* =0.252).

**Conclusions:**

Implementing a sedation protocol that targeted light sedation and reduced benzodiazepine use led to significant improvements in the duration of mechanical ventilation and the requirement for tracheostomy, despite increases in the prevalence and duration of ICU delirium.

## Introduction

Pain, agitation and delirium are common occurrences in critically ill patients, with potential untoward consequences, and they often necessitate treatment with analgesic, sedative and antipsychotic medications. Over the last 15 years, considerable evidence has accumulated demonstrating that both choice of agent and how we use these drugs can significantly impact clinically relevant patient outcomes. This, in turn, has influenced recent pain, agitation and delirium guidelines [1]. With respect to the use of sedatives, guidelines recommend targeting light levels of sedation, daily interruption of sedation, use of a valid and reliable sedation assessment tool and optimizing sedation practices with a multidisciplinary approach to education, protocol development and clinical practice. Different from previous recommendations [2], these guidelines suggest a shift away from benzodiazepines as the mainstay of therapy in favor of non-benzodiazepine alternatives, including dexmedetomidine or propofol.

Some studies have shown that, compared with benzodiazepines, dexmedetomidine may reduce the prevalence of coma and/or delirium and increase days free of delirium [3,4]. However, some researchers have reported conflicting results of randomized controlled trials in which delirium was assessed using less rigorous, unvalidated criteria [5,6]. Two randomized controlled trials have also demonstrated a reduced duration of mechanical ventilation compared with a continuous infusion of midazolam [4,6]. The utility of dexmedetomidine may be limited, however, by increased incidences of hypotension and bradycardia, limited ability to achieve deep sedation (when indicated) and higher drug cost. Propofol has also been shown to reduce the duration of mechanical ventilation compared with benzodiazepines [7], whereas the impact on delirium is not well described. Unique concerns with regard to propofol include hypertriglyceridemia, pancreatitis and propofol infusion syndrome, in addition to the potential for hypotension and respiratory depression.

As a result of many sedative-specific issues that may limit appropriateness or tolerance in certain patient populations, it is often necessary to have multiple agents available for use when managing patients in the intensive care unit (ICU) with a broad array of acute and chronic disease states. It therefore becomes less clear how to apply the findings of randomized controlled trials in clinical practice and to determine how effective such changes might be in this context. Given the potential benefits of dexmedetomidine as compared with benzodiazepines, we sought to assess the clinical effectiveness of modifying our sedation protocol to minimize benzodiazepine use in favor of increased early dexmedetomidine while maintaining other recommended pain, agitation and delirium practices.

## Materials and methods

### Study design

We conducted a before-after study to assess the clinical effectiveness of a sedation protocol minimizing benzodiazepines and favoring early use of dexmedetomidine. The study was approved by the Washington University in St Louis Human Research Protection Office, and waiver of consent was granted.

### Study setting and patient population

This study was conducted at Barnes-Jewish Hospital, a 1,200-bed academic medical center in St Louis, MO, USA. Patients were included if they were mechanically ventilated in the surgical ICU (SICU) or medical ICU (MICU) and required continuous sedation (midazolam, propofol or dexmedetomidine) for at least 24 hours. Exclusion criteria included age less than 18 years, intubation at an outside hospital with prior sedation for >24 hours, need for neuromuscular blockade, norepinephrine infusion at a rate >10 μg/min (or 0.15 μg/kg/min) or use of multiple vasopressors continuously for the initial 24 hours of mechanical ventilation, heart rate <50 beats/min in the 24 hours preceding initiation of continuous sedation, tracheostomy placement prior to hospital admission, history of dementia, acute neurologic injury (acute stroke, seizure or severe traumatic brain injury) or death within 72 hours of intubation.

In the before phase, consecutive eligible patients were enrolled between 1 April 2011 and 31 August 2011. Subsequently, the sedation protocol was modified through a multidisciplinary team approach, with the goal of minimizing use of benzodiazepine infusions and favoring early use of dexmedetomidine. Physician and nursing staff education was provided that emphasized optimizing analgesia prior to initiating sedation, targeting light sedation, using continuous sedation only when absolutely necessary, minimizing benzodiazepine use, proper sedative selection based on patient characteristics, and review of the supporting evidence. The new sedation protocol was implemented on 1 April 2012. The after phase was initiated 1 month later to allow for implementation of the new protocol and included consecutive eligible patients between 1 May 2012 and 31 October 2012.

### Sedation-related practices and protocol

The MICU and SICU both use a nursing-directed sedation protocol whereby the bedside nurses titrate sedatives and analgesics to meet patient-specific goals for level of sedation and pain management, as delineated by the ICU team. In both ICUs, the level of sedation was assessed by the bedside nurse with the Richmond Agitation–Sedation Scale (RASS) and documented at least every 4 hours and as needed for patients requiring mechanical ventilation. The goal level of sedation in both phases was a RASS of 0 to −2, unless an alternative goal was deemed necessary by the ICU team. In the before phase, the predominant sedative used as a continuous infusion was midazolam, although propofol and dexmedetomidine were permitted according to physician discretion. Through a multidisciplinary approach, the sedation protocol was subsequently modified. The only significant change in the scope or structure of the protocol was to recommend dexmedetomidine as the preferred sedative in the absence of patient characteristics that would predict intolerance or for which another sedative might be most appropriate. Specifically, the protocol recommended that dexmedetomidine not be used if any of the following were present: traumatic brain injury, acute and/or uncontrolled seizures, norepinephrine dose >10 μg/min (or 0.15 μg/kg/min), requirement for multiple vasopressors and/or inotropic support, heart rate <50 beats/min during the last 24 hours, sick sinus syndrome, second- or third-degree heart block with no internal pacer, or treatment with continuous neuromuscular blockade. The recommended initial dose of dexmedetomidine was 0.5 μg/kg/hr, and a range of 0.2 to 1.5 μg/kg/hr was allowed. Bolus doses of dexmedetomidine were not recommended. Propofol and midazolam were also allowed in the after phase according to physician discretion. In both phases, bolus doses of midazolam were administered as needed for the management of breakthrough agitation.

Pain was assessed using a visual analogue scale or the Wong-Baker FACES Pain Scale for patients who were able to communicate a response or via an institution-specific non-communicative pain scale for those unable to respond. The non-communicative pain scale assesses facial expression, motor activity, patient vocalizations, caregiver rating of pain, and changes in patient behavior. The pain scale ratings were not collected as a data point. The primary treatment modality for pain in both phases was a fentanyl infusion and/or boluses as needed, according the ICU team’s discretion.

Prior to the before phase, bedside nurses were trained to perform the Confusion Assessment Method for the ICU (CAM-ICU) via an online education module and additional one-to-one instruction at the bedside was provided by clinical nurse specialists dedicated to each ICU. The CAM-ICU assessments were performed twice daily, at approximately 7:00 am and 7:00 pm, and documented accordingly. It was not required that sedation be stopped for the evaluation, and all assessments for patients at a RASS of −3 and higher were included, consistent with the practice in which the CAM-ICU was validated. The delirium treatment section of the protocol was the same in both the before and after phases and provided the option for scheduled and/or as needed intravenous haloperidol to manage hyperactive delirium, defined as a positive CAM-ICU with a RASS of +3 or +4. Alternative antipsychotic agents were also permitted at the discretion of the ICU team.

In both ICUs, a daily spontaneous breathing trial protocol was used to facilitate liberation from the ventilator. In addition, spontaneous awakening was practiced in the SICU by protocol, whereas sedative interruption was performed according to physician order in the MICU.

### Data collection and definitions

Patients were identified for potential inclusion via daily review of the SICU and MICU census Monday through Friday by clinical pharmacists. Data was then retrieved retrospectively via a combination of electronic query of the pharmacy informatics database and manual chart review. Data points collected included baseline demographics and comorbidities; reasons for requiring mechanical ventilation and ICU admission (assessed by intensivists); modified Acute Physiology and Chronic Health Evaluation (APACHE) II score (excluding neurologic assessment); opioid, sedative and antipsychotic use; all CAM-ICU assessments during the ICU stay (up to a maximum of 14 days); all RASS assessments during the period of mechanical ventilation (up to a maximum of 14 days); use of vasopressors and inotropes; and relevant clinical outcomes, including duration of mechanical ventilation, need for reintubation or tracheostomy, and ICU and hospital lengths of stay.

The primary outcomes were the prevalence of delirium and duration of mechanical ventilation. Criteria for delirium were met when patients had a RASS of −3 or higher and a concomitant positive CAM-ICU assessment. Patients were considered liberated from the mechanical ventilator after removal of the endotracheal tube or after 24 hours of continuous tracheostomy collar in those patients requiring tracheostomy. The occurrence of dexmedetomidine failure leading to drug discontinuation was also assessed via manual chart review, and the reason for failure was categorized as inability to achieve RASS goal, hypotension, or bradycardia.

### Statistical analysis

A power analysis was performed *a priori* to determine the minimum sample size necessary to detect clinically relevant reductions in the prevalence of delirium and the duration of mechanical ventilation that were consistent with results from previous randomized controlled trials [4,6]. The baseline prevalence of delirium and duration of mechanical ventilation were estimated based on a retrospective database that included 73 SICU and MICU patients from Barnes-Jewish Hospital meeting the same inclusion criteria. The observed prevalence of delirium was 85%, and mean duration of mechanical ventilation was 138 (±95) hours. In order to detect a 20% reduction in the prevalence of delirium, 75 patients in each group were required at a power of 80% and an α level of 0.05. At the same power and α levels, 100 patients in each group were required to identify a 38-hour reduction in the duration of mechanical ventilation.

All statistical analyses were performed using SAS version 9.3 software (SAS Institute, Cary, NC, USA). Categorical data were compared using a χ^2^ test or Fisher’s exact test. Continuous variables were compared using a Student’s *t*-test, Mann–Whitney *U* test or Wilcoxon rank-sum test, as appropriate. All *P*-values are two-sided and are not adjusted for multiple comparisons. Time to extubation was plotted using Kaplan-Meier survival analysis, and differences between groups were compared with the log-rank test. A Cox proportional hazards analysis was also performed to assess the impact of both treatment phase and APACHE II score on the time to extubation.

## Results

A total of 199 patients were included, with 97 patients in the before phase and 102 in the after phase. The two groups were well balanced with respect to baseline demographics, ICU type, reasons for ICU admission and mechanical ventilation, and severity of illness (Table 1). Baseline comorbidities were also similar between groups with the exception of diabetes mellitus, which was more common in the after group.

**Table 1:** **Patient baseline characteristics** ^**a**^

	**Before phase (n =97)**	**After phase (n =102)**	***P*** **-value**
Age, yr, median (IQR)	58 (43 to 66)	56 (41 to 64)	0.332
Sex, n (%)			0.372
Male	50 (52%)	59 (58%)	
Female	47 (48%)	43 (42%)	
Ethnicity, n (%)			0.196
Caucasian	59 (61%)	57 (56%)	
African American	33 (34%)	37 (36%)	
Other	3 (3%)	0 (0%)	
Not documented	2 (2%)	8 (8%)	
Weight, kg, median (IQR)	83 (66 to 97)	86 (72 to 102)	0.153
Intensive care unit, n (%)			
Surgical/trauma	67 (69%)	61 (60%)	0.173
Medical	30 (31%)	41 (40%)	
Reason for ICU admission, n (%)			
Surgical	34 (35%)	43 (42%)	0.282
Medical	36 (37%)	40 (39%)	
Trauma	27 (28%)	19 (19%)	
APACHE II score,^b^ median (IQR)	21 (17–23)	19 (16–24)	0.800
Vasopressor at baseline, n (%)	53 (55%)	49 (48%)	0.352
Reason for mechanical ventilation, n (%)			
Postoperative	24 (25%)	17 (17%)	0.803
Chest trauma	9 (9%)	6 (6%)	
Pneumonia	11 (11%)	11 (11%)	
Severe sepsis	21 (22%)	18 (18%)	
Cardiogenic pulmonary edema	3 (3%)	5 (5%)	
PE	2 (2%)	3 (3%)	
Asthma	6 (6%)	10 (10%)	
Cardiac arrest	1 (1%)	1 (1%)	
Airway protection	15 (15%)	22 (22%)	
ARDS	3 (3%)	6 (6%)	
Other	2 (2%)	3 (3%)	
Past medical history, n (%)			
Hypertension	49 (51%)	54 (53%)	0.732
Cardiac disease	29 (30%)	31 (30%)	0.939
DM	20 (21%)	36 (35%)	0.021
Asthma or COPD	24 (25%)	32 (31%)	0.299
Malignancy	16 (16%)	17 (17%)	0.974
End-stage renal disease	5 (5%)	6 (6%)	1.0
Cirrhosis	2 (2%)	4 (4%)	0.684
Number of spontaneous breathing trials per patient, median (IQR)	3 (1 to 4)	2 (1 to 5)	0.870

^a^ARDS, Acute respiratory distress syndrome; COPD, Chronic obstructive pulmonary disease; DM, Diabetes mellitus; ICU, Intensive care unit; IQR, Interquartile range; PE, Pulmonary embolism. ^b^Modified Acute Physiology and Chronic Health Evaluation (APACHE) II score excludes neurologic assessment.

### Sedative and opioid use and dosing

The percentage of patients receiving midazolam as the initial sedative was significantly reduced in the before versus after phase (58% versus 27%, *P* <0.0001), as was the percentage of patients receiving a midazolam infusion at any point during their ICU stay (76% versus 48%, *P* <0.0001). Among patients receiving a midazolam infusion, the median total dose and infusion rates were significantly lower in the after phase (Table 2). Dexmedetomidine use as the initial sedative significantly increased in the after phase (2% versus 39%, *P* <0.0001), as did the percentage of patients ever receiving a dexmedetomidine infusion during their ICU stay (39% versus 82%, *P* <0.0001). Propofol use did not differ significantly between phases in either the proportion of patients receiving or the dose administered (Table 2).

**Table 2:** **Sedative and opioid usage and dosing** ^**a**^

	**Before (n =97)**	**After (n =102)**	***P*** **-value**
*Sedative and opioid use*			
Initial sedative, n (%)			
Midazolam	56 (58%)	28 (27%)	<0.0001
Propofol	39 (40%)	34 (33%)	0.315
Dexmedetomidine	2 (2%)	40 (39%)	<0.0001
Second sedative, n (%)			
Midazolam	16 (16%)	17 (17%)	0.834
Propofol	9 (9%)	9 (9%)	
Dexmedetomidine	30 (31%)	37 (37%)	
Not applicable	42 (43%)	38 (38%)	
Received at any time in ICU, n (%)			
Midazolam Infusion	74 (76%)	49 (48%)	<0.0001
Dexmedetomidine	38 (39%)	83 (82%)	<0.0001
Propofol	53 (55%)	49 (48%)	0.396
Fentanyl infusion	97 (98%)	95 (93%)	0.171
*Sedative and opioid dosage and duration*			
Midazolam			
Patients receiving, n (%)	76 (76%)	49 (48%)	
Total dose, median (mg)	143 (91 to 231)	56 (23 to 137)	0.0001
Infusion duration, hr, median (IQR)	58 (29 to 103)	38 (20 to 104)	0.06
Infusion rate, mg/kg/hr, median (IQR)	0.03 (0.02 to 0.05)	0.02 (0.01 to 0.04)	0.0124
Dexmedetomidine			
Patients receiving, n (%)	38 (39%)	84 (82%)	
Total dose, mg, median (IQR)	1.8 (0.6 to 3.1)	2.2 (0.6 to 5.0)	0.541
Infusion duration, hr, median (IQR)	36 (29 to 65)	41 (26 to 85)	0.365
Infusion rate, μg/kg/hr, median (IQR)	0.51 (0.35 to 0.82)	0.57 (0.35 to 0.89)	0.921
Propofol			
Patients receiving, n (%)	53 (55%)	50 (48%)	
Total dose, g, median (IQR)	4.78 (2.03 to 7.27)	3.78 (1.94 to 8.93)	0.797
Infusion duration, hr, median (IQR)	33 (14 to 61)	33 (15 to 54)	0.982
Infusion rate, μg/kg/min, median (IQR)	29 (20 to 42)	29 (28 to 43)	0.981
Fentanyl			
Patients receiving, n (%)	97 (98%)	95 (93%)	
Total dose, mg, median (IQR)	7.85 (4 to 20.3)	4.10 (2.3 to 14.7)	0.002
Infusion duration, hr, median (IQR)	84 (36 to 147)	54 (33 to 128)	0.078
Infusion rate, μg/kg/hr, median (IQR)	1.5 (0.8 to 2.4)	1.1 (0.6 to 1.7)	0.002

^a^ICU, Intensive care unit; IQR, Interquartile range.

A second sedative was used in 56% and 63% of patients in the before and after phase, respectively, either as a result of a switch or as an adjunct. Use of second sedative was most commonly the result of a switch in sedative agent (78% versus 71%) as opposed to being used as an adjunct (22% versus 29%) in both the before and after phases. Choice of second sedative agent did not differ significantly between phases (Table 2). Among patients receiving dexmedetomidine as the second agent (n =67), 63% (n =42, before =22, after =20) initially received midazolam and 37% (n =25, before =8, after =17) initially received propofol. The median time to initiation of dexmedetomidine as a second agent was 39 (IQR, 15.75 to 80.0) hours. Fentanyl infusions were used to treat pain in a high percentage of patients in both groups (98% versus 93%, *P* =0.171). Median total fentanyl dose was significantly lower in the after phase (7.85 mg (IQR, 4 to 20.3) versus 4.10 mg (IQR, 2.3 to 14.7), *P* =0.0019).

### Level of sedation and delirium

The median percentage of RASS scores, per patient, at the goal level of sedation (RASS 0 to −2) was not significantly different in the before versus after phase (Table 3). The median percentage of scores above goal (RASS +1 to +4) was higher in the after phase (5% (IQR 1 to 11) versus 9.5% (IQR 3 to 19), *P* =0.001), whereas the percentage of scores at a moderate level of sedation (RASS −3) was significantly reduced (30% (IQR 15 to 43) versus 18% (IQR 10 to 46), *P* =0.049). No difference was observed in the median percentage of scores at deep sedation (RASS −4 to −5) (Table 3).

**Table 3:** **Level of sedation and delirium assessment** ^**a**^

**Outcome**	**Before (n =97)**	**After (n =102)**	***P*** **-value**
Level of Sedation			
Percentage of RASS scores +4 to +1	5% (1 to 11)	9.5% (3 to 19)	0.001
Percentage of RASS scores +4	0% (0 to 0)	0% (0 to 0)	
Percentage of RASS scores +3	0% (0 to 0)	0% (0 to 0)	
Percentage of RASS scores +2	0% (0 to 2.7)	2.3% (0 to 5.4)	
Percentage of RASS scores +1	3.7% (0.9 to 7.6)	5.3% (1.5 to 12.5)	
Percentage of RASS scores 0 to −2	45% (24 to 64)	48% (20 to 68)	0.789
Percentage of RASS scores −3	30% (15 to 43)	18% (10 to 36)	0.049
Percentage of RASS scores −4 to −5	13% (5 to 23)	9% (2 to 24)	0.154
Delirium evaluation^b^			
Number of potential evaluations	1564	1,585	
Number of evaluations performed	1,415 (90%)	1,475 (93%)	
Number of evaluations NOT performed	149 (10%)	110 (7%)	
Number deemed unassessable^c^	161/1,415 (11%)	262/1,475 (18%)	
Number of positive CAM-ICU assessments	693/1,415 (49%)	806/1,475 (55%)	
Number of negative CAM-ICU assessments	561/1,415 (40%)	407/1,475 (27%)	
Delirium^b^			
Delirium at any time during ICU	79 (81%)	95 (93%)	0.013
Percentage of days with delirium	55% (18 to 83)	71% (45 to 100)	0.001
Median number of positive CAM-ICU assessments	6 (1.5 to 11)	5.5 (3 to 12.25)	0.312
Received antipsychotic medication^d^	28 (29%)	31 (30%)	0.814

^a^CAM-ICU, Confusion Assessment Method for the intensive care unit; RASS, Richmond Agitation–Sedation Scale. ^b^Delirium was assessed by using the CAM-ICU and was performed by the bedside nurse once per shift (every 12 hours) during the ICU stay, up to 14 days. ^c^Patients were unassessable if RASS was −4 or −5 at the time of the evaluation. ^d^Includes any typical or atypical antipsychotic administered during the ICU stay, up to 14 days. All values reported are either n (%) of patients or median (IQR).

The median number of CAM-ICU assessments per patient was 14 (IQR, 9 to 20) in the before phase and 11 (IQR, 6 to 17) in the after phase (*P* =0.007). In the before phase, 10% of the potential CAM-ICU assessments were not performed, and patients were deemed unassessable for 11% of the evaluations performed. In the after phase, 7% of the potential CAM-ICU assessments were not performed, and patients were deemed unassessable for 18% of the evaluations performed (Table 3). Both the prevalence (81% versus 93%, *P* =0.013) and median percentage of days with delirium (55% (IQR, 18 to 83) versus 71% (IQR, 45 to 100), *P* =0.001) were increased in the after phase. Antipsychotics were administered to 29% and 30% of patients in the before and after groups, respectively (*P* =0.814).

### Clinical outcomes

**Figure 1:**
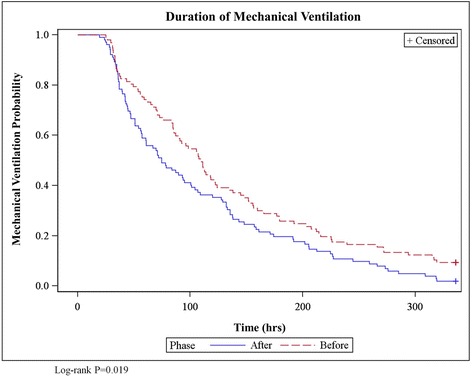
**Duration of mechanical ventilation.** Duration of mechanical ventilation was calculated as the time from intubation until successful removal of the endotracheal tube or after 24 hours of continuous tracheostomy collar in patients requiring tracheostomy. Patients remaining on mechanical ventilation longer than 14 days were censored after day 14.

The median duration of mechanical ventilation was significantly reduced in the after phase (110 (IQR, 59 to 192) hours versus 74.5 (IQR, 42 to 148) hours, *P* =0.029). Time to extubation is displayed in Figure 1. In the Cox proportional hazards analysis of time to extubation, the treatment phase was significant (HR, 1.42 (95% confidence interval (CI), 1.06 to 1.90), *P* =0.019), whereas APACHE II score was not (HR, 0.95 (95% CI, 0.96 to 1.01), *P* =0.239). Significantly fewer patients required tracheostomy in the after phase (20% versus 9%, *P* =0.040). There were no significant differences between phases in the occurrence of self-extubation (3% versus 5%, *P* =0.721) or the need for reintubation (6% versus 13%, *P* =0.149).

The median ICU length of stay was 8 days (IQR, 4 to 12) in the before phase and 6 days (IQR, 3 to 11) in the after phase (*P* =0.252). Median hospital length of stay was not different between groups (17 (IQR, 12 to 23) versus 16.5 (IQR, 10 to 25) days, 0.770). Mortality occurred in eight and nine patients in the before and after phases, respectively (*P* =1.0) (Table 4).

**Table 4:** **Clinical outcomes** ^**a**^

**Outcome**	**Before (n =97)**	**After (n =102)**	***P*** **-value**
Duration of mechanical ventilation, hr, median (IQR)	110 (59 to 192.3)	74.5 (42.3 to 148)	0.029
ICU length of stay, days, median (IQR)	8 (4 to 12)	6 (3 to 11)	0.252
Hospital length of stay, days, median (IQR)	17 (12 to 23)	16.5 (10 to 25)	0.770
Required reintubation, n (%)	6 (6%)	13 (13%)	0.149
Self-extubation, n (%)	3 (3%)	5 (5%)	0.721
Required tracheostomy, n (%)	19 (20%)	9 (9%)	0.040
Mortality, n (%)	8 (8%)	9 (9%)	1.0

^a^ICU, Intensive care unit; IQR, Interquartile range.

### Adverse events

At baseline, 55% and 48% of patients in the before and after phases, respectively, required vasopressor support (*P* =0.352). No significant differences were observed between the before and after phases in the percentage of patients requiring a vasopressor dose increase (≥5 μg/min of norepinephrine or ≥50 μg/min of phenylephrine) or in the occurrence of bradycardia (heart rate <50 beats/min) while the patients were receiving continuous sedation (Table 5). Significantly fewer patients required the addition of a vasopressor in the after phase (26% versus 10%, *P* =0.003).

**Table 5:** **Potential adverse effects**

**Outcome**	**Both phases combined (n =199)**	**Before (n =97)**	**After (n =102)**	***P*** **-value** ^**a**^
Addition of vasopressor, n (%)	35	25 (26%)	10 (10%)	0.0031
While on midazolam	20 (57%)	16 (16%)	4 (4%)	
While on propofol	7 (20%)	7 (7%)	0	
While on dexmedetomidine	6 (17%)	2 (2%)	4 (4%)	
While receiving multiple sedatives	1 (3%)	0	1 (1%)	
While off sedation^b^	1 (3%)	0	1 (1%)	
Increased dose of vasopressor,^c^ n (%)	45	23 (24%)	22 (22%)	0.718
While on midazolam	21 (47%)	17 (18%)	4 (4%)	
While on propofol	10 (22%)	4 (4%)	6 (6%)	
While on dexmedetomidine	10 (22%)	2 (2%)	8 (8%)	
While receiving multiple sedatives	1 (2%)	0	1 (1%)	
While off sedation^b^	3 (7%)	0	3 (3%)	
Addition of inotrope, n (%)	9	6 (6%)	3 (3%)	0.151
While on midazolam	5 (56%)	3 (3%)	2 (2%)	
While on propofol	3 (33%)	3 (3%)	0	
While on dexmedetomidine	1 (1%)	0	1 (1%)	
While receiving multiple sedatives	0	0	0	
While off sedation^b^	0	0	0	
Heart rate <50 beats/min, n (%)	25	14 (14%)	11 (11%)	0.438
While on midazolam	5 (20%)	4 (4%)	1 (1%)	
While on propofol	9 (36%)	6 (6%)	3 (3%)	
While on dexmedetomidine	7 (28%)	3 (3%)	4 (4%)	
While receiving multiple sedatives	2 (8%)	1 (1%)	1 (1%)	
While off sedation^b^	2 (8%)	0	2 (2%)	

^a^ *P*-value is for the before versus after phase comparison. ^b^Potential adverse effect occurred during a period of time when no continuous sedation was being administered (for example, during a sedative interruption or patient may have only been receiving analgesia at that time). ^c^Increased dose was defined as an increase from baseline dose of ≥5 μg/min of norepinephrine or ≥50 μg/min for phenylephrine.

Patients may have been receiving any of the three sedatives at the time of a potential adverse event. Table 5 details which sedative was being administered at the time of the event. With respect to the need for initiation of or increased dose of a vasopressor and also the addition of an inotrope, the majority of patients were receiving midazolam. Bradycardia, however, more commonly occurred while patients were receiving propofol or dexmedetomidine.

Dexmedetomidine failure occurred in a total of 30 (25%) of the 121 patients receiving the agent in either the before (n =9) or after (n =21) phase. The reasons for failure were hypotension in 15 patients, inability to achieve RASS goal in 8 patients, and bradycardia in 7 patients.

## Discussion

The results of the present study demonstrate that implementation of a protocol that reduces benzodiazepine use and targets light sedation can lead to significant improvements in the time spent at moderate to deep levels of sedation, the duration of mechanical ventilation, and the requirement for tracheostomy. These benefits were observed despite increases in the prevalence and duration of ICU delirium. Although the primary change between phases was to favor earlier and more frequent use of dexmedetomidine, it is important to note that the sedation protocol allowed use of other sedatives according to the ICU team’s discretion and incorporated other key elements from recent pain, agitation and delirium guidelines, such as goal-directed analgesia therapy, multidisciplinary involvement, use of validated tools for routine assessment of sedation and delirium, and daily spontaneous breathing trials. This study therefore represents a valuable assessment of the clinical effectiveness of modifying sedation practices in a real-world setting.

The observed shift in sedative use indicates that the protocol was effective in reducing midazolam use in favor of early dexmedetomidine as the primary change in sedation-related practices between phases. In parallel with the changes in sedative use, a reduction in sedative assessments at moderate sedation was observed, as well as a small increase in the percentage of scores above goal. Although there is additional room for improvement, this represents a positive step toward the goal of minimizing time spent at moderate to deep levels of sedation. The occurrences of self-extubation and need for reintubation were infrequent and not statistically different between groups; however, it should be noted that both were increased in the after phase, and this study was not sufficiently powered to assess these differences. Interestingly, although more than 90% of patients in both groups received a fentanyl infusion for pain, the total dose was significantly reduced in the after phase. The most likely explanation is a reduced duration of fentanyl infusion secondary to reduced time on mechanical ventilation, although it is possible that the analgesic properties of dexmedetomidine or perhaps an improved ability to assess pain in the after phase could also account for some of this difference. These results are consistent with early randomized trials of dexmedetomidine in surgical patients, which demonstrated reduced opioid doses [8,9]. However, more recent studies examining long-term dexmedetomidine use in mixed medical-surgical ICU populations have shown either no difference [4,6] or increased opioid needs compared with benzodiazepines [3]. In the latter study, the authors found that the increased opioid needs were primarily in patients targeted for deep sedation, and they proposed that this likely reflected clinicians’ use of fentanyl to achieve deeper sedation.

With respect to potential adverse effects, increased occurrences of hypotension or bradycardia were not observed between groups, and the percentage of patients requiring the addition of a vasopressor while on sedative therapy was actually lower in the after phase. The majority of patients requiring initiation or an increased dose of vasopressor were receiving midazolam at the time of the potential adverse effect. In part, this may have been a result of clinicians choosing to defer the use of propofol and/or dexmedetomidine while patients remained hypotensive. Bradycardia (HR <50 bpm) more commonly occurred while patients were receiving propofol or dexmedetomidine. Dexmedetomidine failure occurred in one in four patients, with the most common reason being hypotension. Although the definitions of hypotension and bradycardia vary among studies, both toxicities have been well described in randomized trials [3-6]. Taken together, these findings suggest that clinicians observed hypotension or bradycardia early and intervened before it reached a concerning level. Failure to achieve RASS goal occurred in 7% of patients, which is slightly lower than the rates of dexmedetomidine discontinuation due to lack of efficacy in the Dexmedetomidine versus Midazolam for Continuous Sedation in the Intensive Care Unit (MIDEX; ClinicalTrials.gov Identifier: NCT00481312) (9%) and Dexmedetomidine Versus Propofol for Continuous Sedation in the Intensive Care Unit (PRODEX; ClinicalTrials.gov Identifier: NCT00479661) (14%) trials [6].

Researchers in multiple studies have reported significant improvements in patient outcomes when sedation is effectively minimized by using various strategies [10-15]. Compared with benzodiazepines, dexmedetomidine has also been shown to improve clinical outcomes, including reduced duration of mechanical ventilation [4,6]. In the present study, a nursing staff-directed sedation protocol targeting light sedation was the standard of care. Daily spontaneous breathing trails were also routinely performed in both the SICU and MICU, and interruption of sedatives was combined with this in the SICU. Similarly to the aforementioned studies comparing continuous infusions of dexmedetomidine and midazolam, we found a significant reduction in time on the ventilator. This finding supports the clinical effectiveness of the protocol and is striking, considering that 27% of patients in the after phase still received midazolam as the initial sedative, which would be expected to reduce between-group differences. One important distinction in our study is that dexmedetomidine was used up front in 39% of patients in the after phase, whereas in randomized controlled trials of dexmedetomidine, the study drug was not initiated until 22 to 41 hours after initiation of mechanical ventilation and prior sedative therapy [3,4]. Delayed initiation of dexmedetomidine in these trials may reduce possible benefits because of residual effects of previous sedatives, particularly prior benzodiazepines. A statistically significant reduction in ICU length of stay was not found, although a trend in favor of the after phase can be appreciated. Individual studies of dexmedetomidine also have not shown a significant reduction in ICU length of stay [3-6], although the authors of one meta-analysis reported this finding [16].

With regard to our hypothesis that reduced midazolam use would lead to reduced prevalence and duration of delirium, we found the opposite. However, this is not entirely inconsistent with the available evidence. In one large study comparing continuous infusions of dexmedetomidine and midazolam, a significant reduction in the prevalence and duration of delirium was observed [4]. When compared with continuous lorazepam, the prevalence of delirium or coma as a combined endpoint was reduced; however, when the individual endpoints were assessed, this difference was found to be primarily a result of reduced coma [3]. In a subsequent subgroup analysis of the same study, the daily risk of delirium was found to be significantly lower in the dexmedetomidine versus the lorazepam group [17]. In a randomized pilot study, Ruokonen and colleagues [5] compared midazolam or propofol to dexmedetomidine and found that the combined endpoint of CAM-ICU and adverse events of delirium and confusion were increased in the dexmedetomidine group (44% versus 25%, *P* =0.035), whereas the proportion of positive CAM-ICU assessments was not different between groups. Finally, in the MIDEX study, delirium was not routinely assessed with a validated tool while patients were receiving sedative therapy, although the trialists reported no difference in a combined endpoint of neurocognitive adverse events (anxiety, agitation or delirium) between the dexmedetomidine and midazolam groups [6].

Similarly to earlier studies including comparable patients [18-22], we found that the overall prevalence of ICU delirium was very high in the present study. Recent studies shed greater light into the diagnosis of ICU delirium as it relates to both the level of sedation and its potential impact on patient outcomes [22,23]. Patel and colleagues [22] performed a prospective cohort study of 102 adult MICU patients requiring mechanical ventilation and continuous infusion of sedatives in which they performed CAM-ICU assessments before and for up to 2 hours after a daily interruption of sedatives and analgesics. The prevalence of delirium was 89% when assessed prior to daily interruption, as opposed to 77% when assessed after interruption. Patients with rapidly reversible, sedation-related delirium had significantly fewer ventilator days and hospital days than did patients who had delirium that persisted after 2 hours. Given that all patients in our present study required continuous sedatives for at least 24 hours and that CAM-ICU assessments were not performed during an interruption of sedative therapy, it is likely that a percentage of this delirium was sedation-related and potentially rapidly reversible, which would explain our findings of reduced duration of mechanical ventilation despite increased delirium. Similarly, Haenggi and colleagues [23] found that the prevalence of delirium (using both CAM-ICU and Intensive Care Delirium Screening Checklist) was reduced by 22% when patients with a RASS of −2 or −3 were excluded, suggesting that the depth of sedation can have a significant influence on the apparent prevalence of delirium. Notably, we included all assessments for patients at a RASS of −3 and higher, consistent with the practice in which the CAM-ICU was originally validated. Despite the reduction in the median percentage of scores at a RASS of −3, the prevalence of delirium was still increased in the after phase. Accordingly, it would seem unlikely that this is an explanation for the increased delirium we observed in the after phase. However, though we observed a reduction in the percentage of scores at moderate sedation, this does not mean that the level of sedation was lower at the time CAM-ICU assessments were performed, as sedation was assessed much more frequently than delirium, which was assessed only twice daily at fixed time points. In fact, the number of CAM-ICU evaluations deemed unassessable was higher in the after phase, suggesting a higher frequency of deep sedation at the time of CAM-ICU assessments. It may thus still be possible that the level of sedation may have influenced the CAM-ICU assessments and the prevalence of delirium. Finally, because use of any combination of all three sedatives was allowed in both phases, it is not possible in the present before-after open-label trial to truly discern the relative contribution of each sedative to the observed prevalence of delirium.

Given that other important outcomes, such as time on mechanical ventilation, were improved in the after phase, and no evidence that the increased delirium in the after phase produced harm, an inference that is potentially just as important to note is that delirium *per se*, as measured by CAM-ICU in clinical practice, may not always reliably predict other important quality and safety indicators and may not alone be a sufficient basis on which to change practice. Despite the increased delirium we identified in our cohort, we have not changed our practice back to the earlier benzodiazepine-based sedation protocol, given the observed benefits in regard to other important clinical outcomes. Perhaps production of sedation-related, rapidly reversible delirium may not be as negative, as long as it does not result in more prolonged ventilation or other adverse events. This requires further study, given the associations between delirium and a negative impact on both short- and long-term outcomes.

This study has several important limitations. First, rather than one discrete change in drug therapy, this study assesses the impact of implementing a sedation protocol that merely favors the early use of dexmedetomidine to minimize use of midazolam infusions. Use of alternative sedatives was still allowed in both phases. Additionally, we provided reeducation of sedation-related principles to facilitate implementation of the protocol. Therefore, it is difficult to establish that the observed differences are due solely to the change in sedative therapy. However, this approach provides useful insight into the clinical effectiveness of such a change in an ICU clinical practice, where the use of multiple sedatives is often necessary.

Second, pain was assessed using an institution-specific scale for non-communicative patients that has not been validated, and pain assessments were not collected. However, frequent assessments of pain and appropriate management were emphasized in both ICUs, and patients appeared to be treated aggressively, with over 90% of patients in both phases receiving continuous infusions of fentanyl. Third, although all nurses were required to complete an online education module and one-to-one follow-up by clinical nurse specialists dedicated to the SICU and MICU was provided, CAM-ICU assessments were performed by the bedside nurse and were not validated by other practitioners. Therefore, it is possible that some inaccuracy exists related to interrater reliability and changes in assessments and scoring that may have occurred as the study progressed. Nevertheless, this is representative of how delirium is commonly assessed in clinical practice and thus makes our results more generalizable to typical ICU environments.

With respect to the assessment of sedative failure, we chose to focus on the potential failure of dexmedetomidine because (1) a main goal of the protocol was to increase dexmedetomidine use and (2) less is known about tolerance in a real-world setting. Although we report potential hypotension and bradycardia events for all drugs, we did not assess for midazolam and propofol failure in the same way. Finally, this was a before-after study and was not blinded or randomized, and thus a risk of bias cannot be eliminated.

## Conclusions

This study demonstrates the clinical effects of implementing a sedation protocol aimed at minimizing benzodiazepine use in favor of early use of dexmedetomidine while maintaining other best practices with respect to pain, agitation and delirium. Despite an increased prevalence and duration of delirium, the level of sedation and duration of mechanical ventilation were improved. Although the impact on duration of mechanical ventilation was positive and consistent with previous studies, further investigation of the impact of CAM-ICU-identified delirium in the real-world setting is warranted. It is also important to note that dexmedetomidine intolerance may occur in one of four patients, owing to lack of efficacy, hypotension or bradycardia.

## Key messages

- The new protocol was effective in reducing use of continuous benzodiazepine infusions and increasing the use of dexmedetomidine.
- The new protocol resulted in lighter levels of sedation and reduced duration of mechanical ventilation.
- The new protocol increased the prevalence and percentage of ICU days with delirium.
- One in four patients may experience lack of efficacy in achieving desired sedation or intolerance of dexmedetomidine as a result of hypotension or bradycardia.

## Abbreviations

APACHE: Acute Physiology and Chronic Health Evaluation
ARDS: Acute respiratory distress syndrome
CAM-ICU: Confusion Assessment Method for the intensive care unit
CI: Confidence interval
COPD: Chronic obstructive pulmonary disease
HR: Hazard ratio
ICU: Intensive care unit
IQR: Interquartile range
MICU: Medical intensive care unit
MIDEX: Dexmedetomidine versus Midazolam for Continuous Sedation in the Intensive Care Unit
RASS: Richmond Agitation–Sedation Scale
SICU: Surgical intensive care unit
